# Exploring inflammatory bowel disease therapy targets through druggability genes: a Mendelian randomization study

**DOI:** 10.3389/fimmu.2024.1352712

**Published:** 2024-04-18

**Authors:** Shuangjing Zhu, Yunzhi Lin, Zhen Ding

**Affiliations:** Department of Hepatobiliary Surgery, Chaohu Hospital of Anhui Medical University, Hefei, China

**Keywords:** inflammatory bowel disease, ulcerative colitis, Crohn’s disease, Mendelian randomization, therapeutic target

## Abstract

**Background:**

Inflammatory bowel disease is an incurable group of recurrent inflammatory diseases of the intestine. Mendelian randomization has been utilized in the development of drugs for disease treatment, including the therapeutic targets for IBD that are identified through drug-targeted MR.

**Methods:**

Two-sample MR was employed to explore the cause-and-effect relationship between multiple genes and IBD and its subtypes ulcerative colitis and Crohn’s disease, and replication MR was utilized to validate this causality. Summary data-based Mendelian randomization analysis was performed to enhance the robustness of the outcomes, while Bayesian co-localization provided strong evidential support. Finally, the value of potential therapeutic target applications was determined by using the estimation of druggability.

**Result:**

With our investigation, we identified target genes associated with the risk of IBD and its subtypes UC and CD. These include the genes GPBAR1, IL1RL1, PRKCB, and PNMT, which are associated with IBD risk, IL1RL1, with a protective effect against CD risk, and GPX1, GPBAR1, and PNMT, which are involved in UC risk.

**Conclusion:**

In a word, this study identified several potential therapeutic targets associated with the risk of IBD and its subtypes, offering new insights into the development of therapeutic agents for IBD.

## 1 Introduction

Inflammatory bowel disease (IBD) is a chronic inflammatory disease of the intestine represented by Crohn’s disease (CD) and ulcerative colitis (UC), which seriously affect the quality of life and health of millions of people worldwide (1). As newly industrialized countries experience economic growth and lifestyle changes, factors such as diet, environmental exposures, and genetic inheritance may be closely linked to the risk of developing IBD (2). By 2025, it is predicted that newly industrialized countries may have a higher number of people with IBD compared to the Western world. This trend has elevated IBD to an important global public health issue (3).

IBD symptoms encompass intestinal manifestations such as diarrhea, blood in the stool, and abdominal pain, greatly impacting patients’ daily life and overall quality of life (4). Furthermore, IBD can lead to complications including malnutrition, cardiovascular diseases, liver and biliary tract disorders, and other intestinal manifestations, posing significant risks to human health (5). Currently, IBD is mostly treated through drug regimens, with commonly used therapeutic agents including anti-inflammatory drugs, immunosuppressants, and biologics (6). Yet, these drugs have possible side effects and are not consistently effective in a subset of patients (7). Firstly, for instance, the anti-inflammatory drug 5-aminosalicylic acid is a preferred treatment for mild-to-moderate UC disease, but an adverse gastrointestinal reaction such as nausea and vomiting can occur with them (7). Second, the biologic agent infliximab (IFLX) treats IBD by hastening the death of pro-inflammatory cells, while prolonged use of IFLX may lead to the possibility of infection, allergy, and even malignancy (8). Third, the immunomodulator azathioprine increases the potential for hepatotoxicity and pancreatitis (9). Hence, the quest for new therapeutic strategies and targets is becoming an ongoing important direction in IBD research.

Genome-wide association studies (GWAS) uncover disease-associated single nucleotide polymorphisms (SNPs) that enable scientists to pinpoint associations between genetic variants and specific diseases, which can be used to aid in the identification and validation of drug targets (10). Nevertheless, disease-causing genes cannot be fully characterized by GWAS analysis alone. Mendelian randomization (MR) is a genetic statistical method for assessing the causality among exposures and outcomes, which minimizes the interference of confounders and reverse cause and effect since genetic variants follow the principle of Mendelian random assignment at the time of conception and are independent of social background and lifestyle (11). Gene expression levels are influenced by genetic variation (eQTL), and cis-expression quantitative trait loci (cis-eQTL) serve as proxies for the gene expansion levels (12). Using drug target MR methods to analyze independent disease abstract GWAS summary datasets and gene cis-eQTL to identify relevant genes causing complex traits (13).

This research aims to contribute to the development of therapeutic targets for IBD by exploring causative genes associated with both the UC and CD subtypes of the disease.

## 2 Materials and methods

### 2.1 Research methods

**Figure 1:**
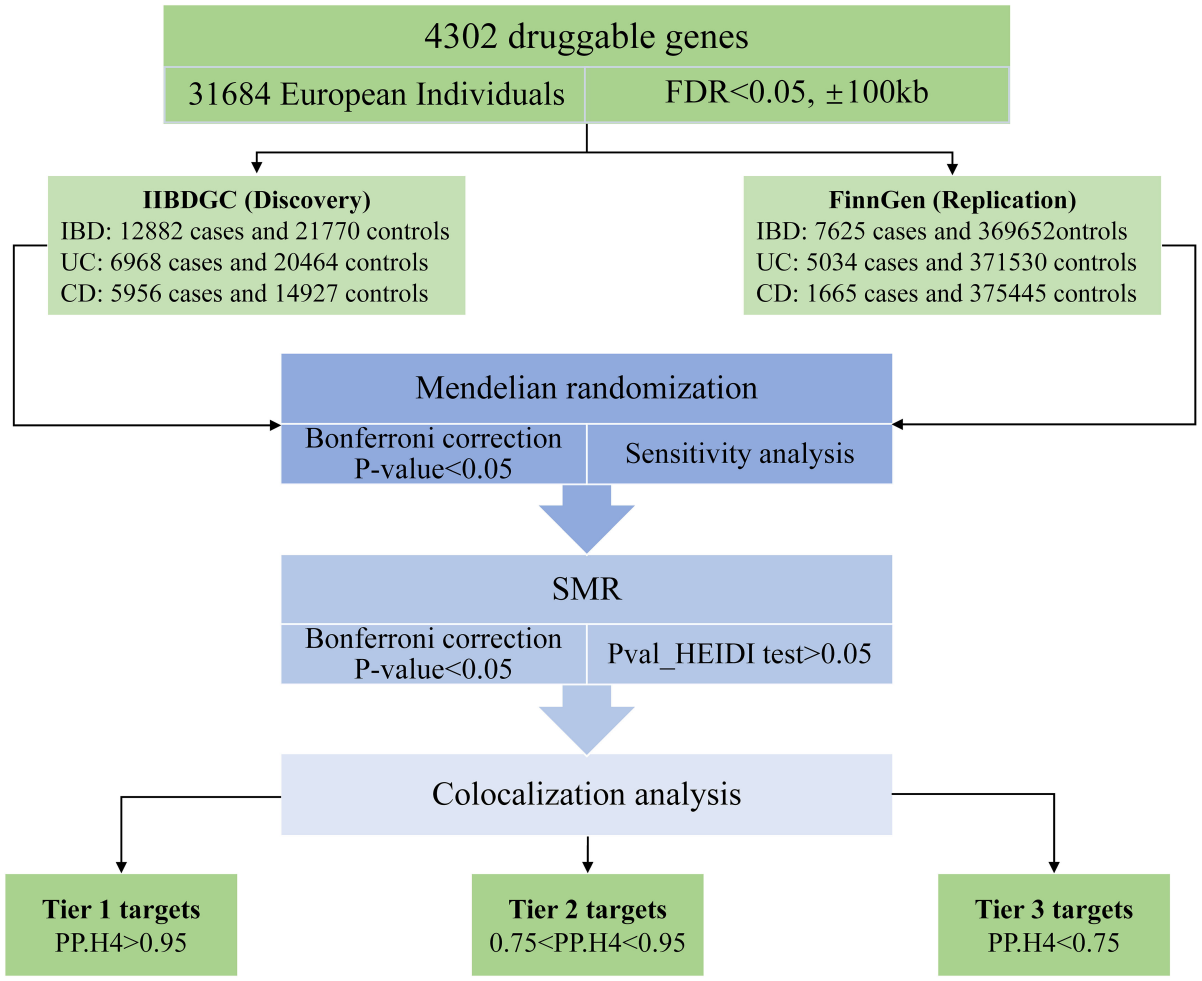
Research design.

The study methods were compliant with the STROBE-MR checklist (14), further details can be found in **Additional File 1**. The study design is depicted in **Figure 1**. In this study, our first step involved utilizing MR methods to evaluate the causal relationship between druggable genes in blood and IBD, encompassing both UC and CD subtypes (15, 16). Additionally, we employed replicated MR, Summary data-based MR analysis (SMR) approaches, along with the heterogeneity in dependent instruments (HEIDI) test, to enhance the robustness of our MR results (12). Subsequently, we conducted a Bayesian co-localization analysis to identify common causal SNPs shared between the genes and the risk of IBD (17). Lastly, we estimated the druggability of the identified genes to investigate their potential as effective therapeutic targets for IBD.

### 2.2 Data sources

Exposure data were extracted from the eQTLGen consortium (https://eqtlgen.org/) and eQTL meta-analysis of peripheral blood samples from 31,684 individuals, with information about 16,987 genes (16). The druggable genes are from a previous study containing a total of 4,479 druggable genes. After removing the null values and genes not located on the autosomes, the number of actionable genes is 4302, We utilized the cis-eQTL for these 4302 genes as exposure (15). IBD GWAS abstract data from the International Inflammatory Bowel Disease Genetics Consortium (IIBDGC, https://www.ibdgenetics.org/), including 12,882 cases of IBD, 6,968 cases of UC, and 5,956 patients of European ancestry with CD (**Table 1**) (18). We also get GWAS summarized data for IBD, UC, and CD from FinnGen’s version R9 database for replication MR analysis (https://r9.finngen.fi/), including 7625 IBD,5034 UC, and 1665 CD patients (**Table 1**). FinnGen Database is a project containing a large number of bio-samples and relevant diagnostic techniques to gather data from national health registries of the Finnish population since 1969. The program aims to delve deeper into the relationship between the genome and health as well as to provide valuable information to the general public health system to promote medical research into the etiology of diseases in the population (19).

**Table 1:** IBD and its subtypes UC and CD data source details.

Disease	Cases	Controls	Population	No SNPs
IBD(IIBDGC)	12882	21770	European	12716084
UC(IIBDGC)	6968	20464	European	12255197
CD(IIBDGC)	5956	14927	European	12276506
IBD(FinnGen)	7625	369652	European	20170236
UC(FinnGen)	5034	371530	European	20170227
CD(FinnGen)	1665	375445	European	20170234

IBD, Inflammatory bowel disease; IIBDGC, International Inflammatory Bowel Disease Genetics Consortium; UC, Ulcerative colitis; CD, Crohn’s disease.

### 2.3 Selection of cis-eQTLs associated with druggable genes

To obtain cis-eQTL data for drug target genes and allele frequency information, only statistically significant cis-eQTLs with an FDR <0.05 were included (13). To generate the genetic instrumental variables used to proxy the 4302 druggable targets, we performed a series of manipulations. First, we chose cis-eQTL within ±100kb from the genomic transcriptional start site, based on the 1000G Genome Europe reference panel setting r2 < 0. 1 to avoid the effect of chain imbalance (20). Second, we carried out a scan in the PhenoScanner database (https://www.phenoscanner.medschl.cam.ac.uk) to delete SNPs that were linked to confounders and IBD, to prevent the interference of confounders (21). Third, to guard against the biasing effects of weak instrumental variables, the F-value statistic was calculated by the formula β²/SE², and when the F-value was less than 10 it would be excluded (22). Finally, the palindromic SNPs might not affect gene expression and protein functions, we will remove those palindromic SNPs with allelic frequencies.

### 2.4 MR analysis

For our MR study, we conducted two-sample MR analyses using cis-eQTL and outcomes. When screened exposures have only one SNP, Wald ratios were applied as the principal analysis method. In cases where there were more than two SNPs, inverse variance weighted (IVW) models were estimated to estimate the effect of each exposure on the outcome, with MR-Egger, MR-RAPS, Maximum likelihood, and Weighted median methods as additional methods (23–25). The genes were only incorporated into the next step of the analysis when four of the five methods were in alignment with each other in the same direction. We deployed Cochran’s Q and MR-Egger intercept tests to examine possible heterogeneity and horizontal pleiotropy of the filtered instrumental variables (26, 27). MR-Steiger was enlisted to assess the potential reverse causality of exposure on outcome (28). When the gene was significant in both the primary MR and replication MR analyses, we proceeded to SMR analysis to further validate the MR results. SMR is the process of using GWAS-level summary data and eQTL to be used for investigating whether there is any causal relationship between one or more genes and specific phenotypes, using HEIDI to test the results (12). SMR software (https://yanglab.westlake.edu.cn/software/smr/) by using the SMR analysis and HEIDI assay. MR analysis using R software TwoSampleMR package (0.5.7) for analysis. We utilize the Bonferroni correction for multiple checks.

### 2.5 Co-localization analysis

We concluded with a Bayesian co-localization analysis of genes that were multiply corrected by MR and SMR. Co-localization analysis combines information from multiple SNPs or other genetic variants to determine whether genes and diseases exist at similar locations in the genome or interact with each other. We use default *a priori* probabilities p1 = 1E-4, p2 = 1E-4, and p12 = 1E-5, representing the likelihood that an SNP in a selected region is associated with significant gene expression, IBD risk, and both. The posterior probabilities were verified against five hypotheses: pp.H0, SNPs were not associated with any of the traits; PP.H1, SNPs were correlated with gene expression but not with IBD risk; PP.H2 were associated with the risk of developing IBD but not with gene expression; PP.H3 were related to both gene expression and IBD risk, but with different causal variants; and PP.H4, were related to IBD risk and gene expression, specifically the same genetic causal variant (17). We set the significance threshold for PP.H4 at 0.95 owing to the limited efficacy of the colocalization assessment. Bayesian co localization was analyzed using the software package coloc (version 5.0.1).

### 2.6 Druggability evaluation

DrugBank (https://go.drugbank.com/) brings together numerous data on the interactions between drugs and genes (29), integrating information from multiple public databases, including drug target prediction, mechanisms of action, and clinical applications to offer vital data and functionality. The potential of identified druggable target genes as therapeutic agents for IBD and its subtypes was further determined by using DrugBank to locate associations between characterized proteins and drugs.

## 3 Results

### 3.1 MR analysis reveals 27 genes associated with IBD, 21 genes associated with UC, and 17 genes associated with CD

**Figure 2:**
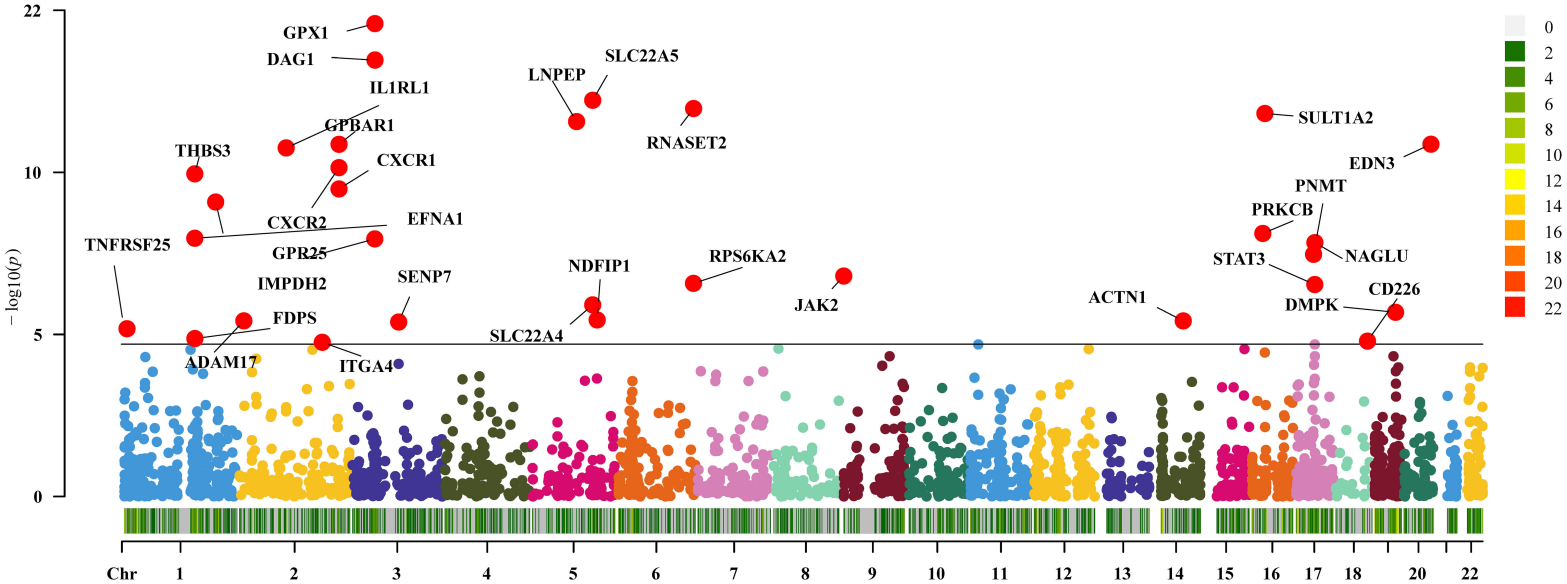
Two-sample Mendelian randomization results for druggable genes and inflammatory bowel disease Manhattan plot.

In the current study cohort, we identified 49 genes with expressions associated with IBD (P<0.05/2641, Bonferroni corrected). Subsequently, in the replication MR analysis, 31 out of these 49 genes exhibited significance in the MR test (P<0.05, **Figure 2**). Sensitivity analysis revealed that the genes SLC22A5, RPS6KA2, and SENP7 did not pass the pleiotropy test (P<0.05), and the gene IMPDH2 did not pass the MR-Sterger test (P>0.05). Furthermore, the genes GPR25, JAK2, STAT3, SLC22A4, and NDFIP1 showed potential heterogeneity.

**Figure 3:**
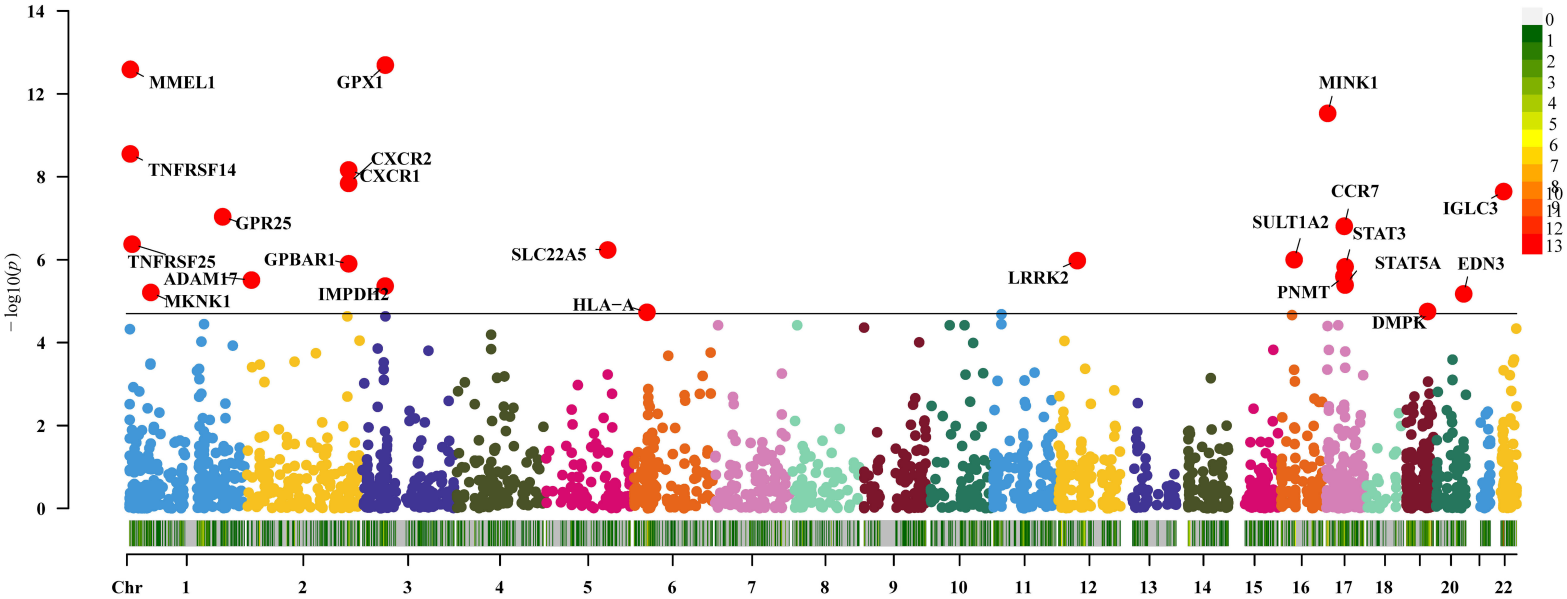
Two-sample Mendelian randomization results for druggable genes and ulcerative colitis Manhattan plot.

The expression of 36 genes was associated with UC (P<0.05/2641, Bonferroni corrected), and subsequent replication MR analysis demonstrated that 23 genes remained significant (P<0.05, **Figure 3**). Sensitivity analysis indicated that the gene SLC22A5 did not pass the pleiotropy test (P<0.05), and the gene IMPDH2 did not pass the MR-Sterger test (P>0.05). Additionally, the genes TNFRSF14 and GPBAR1 exhibited heterogeneity.

**Figure 4:**
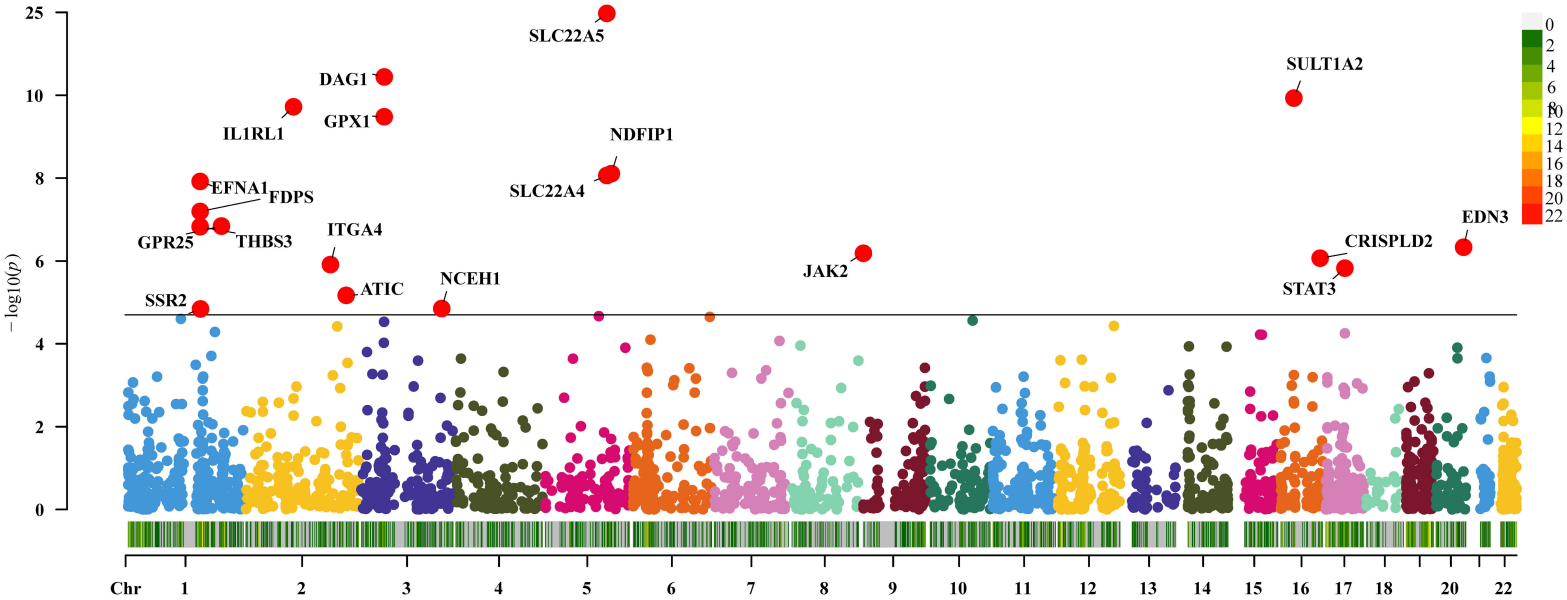
Two-sample Mendelian randomization results for druggable genes and Crohn’s disease Manhattan plot.

Thirty-six genes were causally connected to disease CD by expression (P<0.05/2641, Bonferroni correction). Replication MR showed that 19 of these genes passed the MR test again (P<0.05, **Figure 4**). There was reverse causality for genes DAG1 and SSR2 in the sensitivity analysis (P>0.05) and no pleiotropy (P<0.05). Genes NDFIP1, SLC22A4, THBS3, JAK2, and STAT3 had the presence of heterogeneity. Detailed information on significant gene MR results for IBD and its subtypes UC and CD are shown in **Supplementary Tables 1**-**3**.

### 3.2 SMR analysis validated 7 genes linked to IBD, 4 genes with UC, and 8 genes for CD

**Figure 5:**
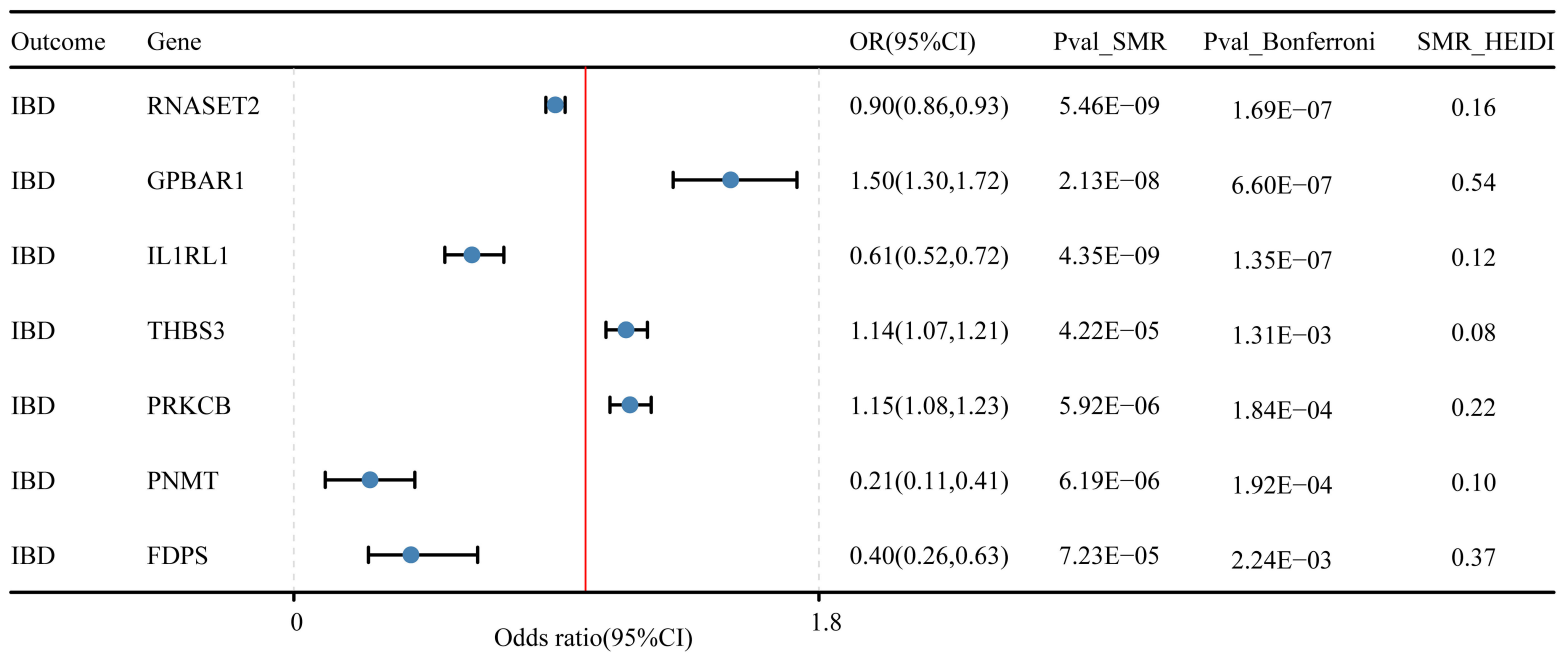
Druggable genes and inflammatory bowel disease SMR results.

**Figure 6:**
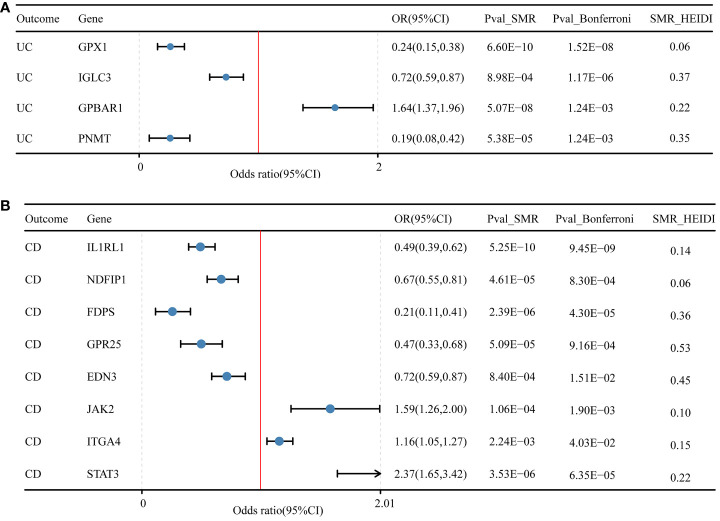
Druggable genes and SMR results for ulcerative colitis and Crohn’s disease. **(A)** Druggable genes and ulcerative colitis SMR results. **(B)** Druggable genes and Crohn’s disease SMR results.

In the discovery cohort and replication cohort MR analyses, an SMR analysis was conducted for the examined genes. A total of 27 genes for IBD, 21 genes for UC, and 17 genes for CD were included in this analysis. Genes that did not pass the HEIDI test and were not consistently oriented (P<0.05, **Figure 5**) were removed from the analysis. As a result, 7 genes showed significant associations with IBD in the SMR analysis (P<0.05/27). For UC, 4 genes passed the SMR analysis (P<0.05/21, **Figure 6A**), and for CD, 8 genes demonstrated significant causal associations (p<0.05/17, **Figure 6B**). **Supplementary Tables 4**-**6** provide detailed information on the SMR analysis.

### 3.3 Identification of genetic overlaps in IBD, UC, and CD using co-localization analysis

**Figure 7:**
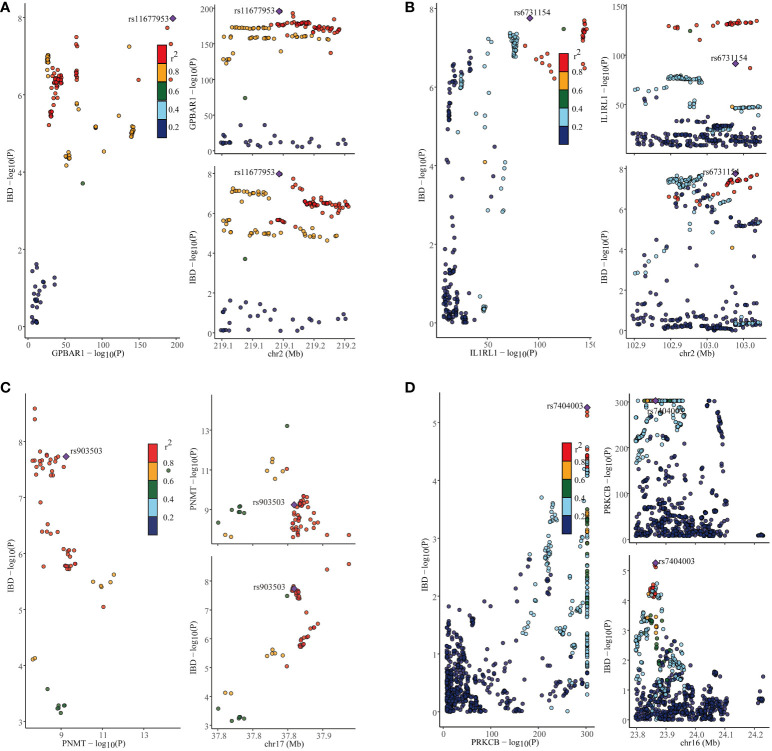
Co-localization results for inflammatory bowel disease. **(A)** Co-localization results of eQTL of the GPBAR1 gene and inflammatory bowel disease. **(B)** Co-localization results of eQTL of the IL1RL1 gene and inflammatory bowel disease. **(C)** Co-localization results of eQTL of the PNMT gene and inflammatory bowel disease. **(D)** Co-localization results of eQTL of the PRKCB gene and inflammatory bowel disease.

**Figure 8:**
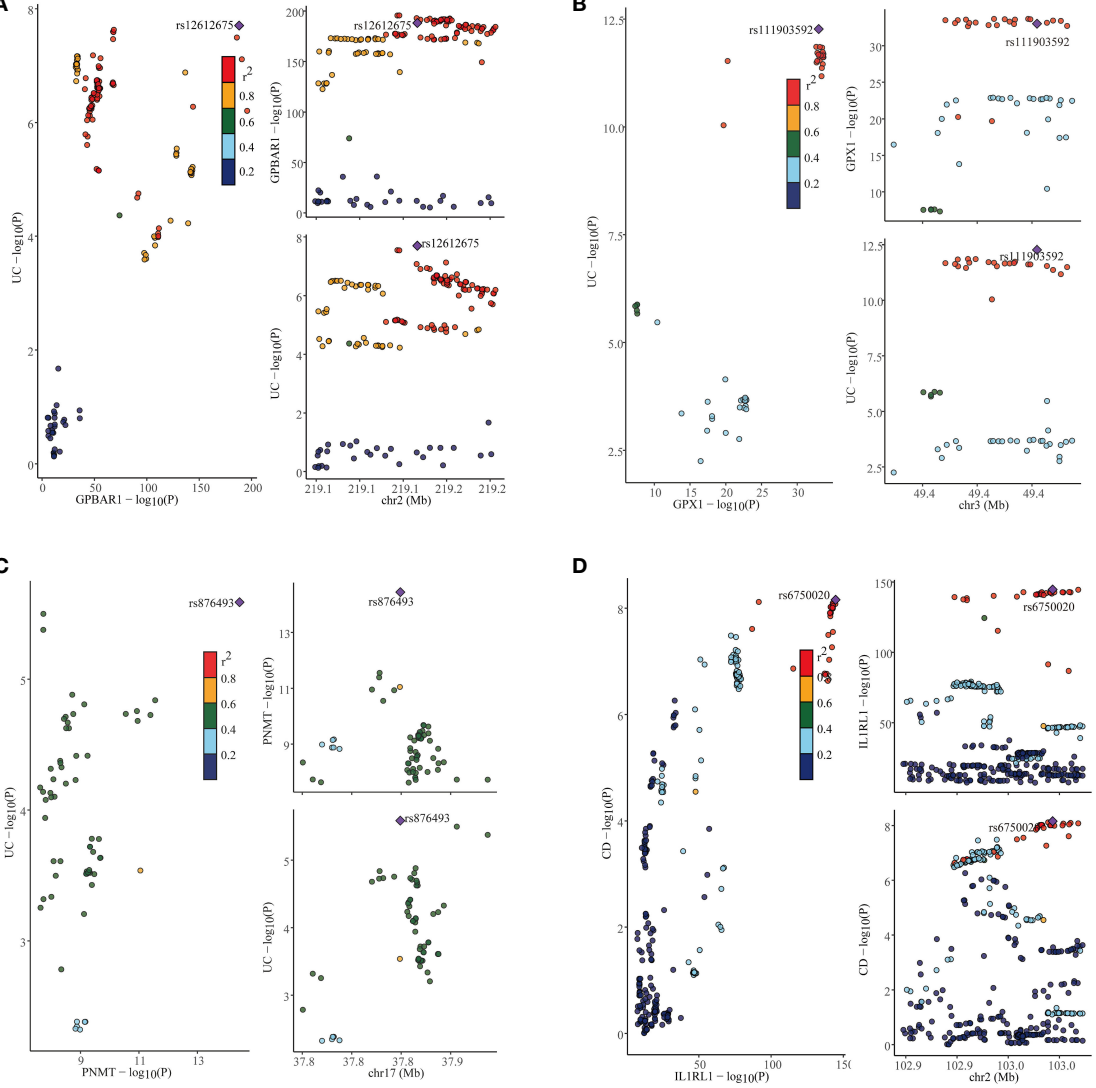
Co-localization results for ulcerative colitis and Crohn’s disease. **(A)** Co-localization results of eQTL of the GPBAR1 gene and ulcerative colitis. **(B)** Co-localization results of eQTL of the GPX1 gene and ulcerative colitis. **(C)** Co-localization results of eQTL of the PNMT gene and ulcerative colitis. **(D)** Co-localization results of eQTL of the IL1RL1 gene and Crohn’s disease.

In our study, we performed the co-localization analysis of the screened genes associated with IBD, UC, and CD (**Table 2**, **Figures 7**, **8**). Our findings revealed that for IBD, genes GPBAR1 (PP.H4 = 0.99), IL1RL1 (PP.H4 = 0.95), PRKCB (PP.H4 = 0.99), and PNMT (PP.H4 = 0.95) exhibited robust evidence of high co-localization support (**Figure 7**). Furthermore, genes GPX1 (PP.H4 = 0.98), GPBAR1 (PP.H4 = 0.99), and PNMT (PP.H4 = 0.99) demonstrated significant co-localization support with UC (**Figure 8**). Additionally, the gene IL1RL1 (PP.H4 = 0.98) showed strong co-localization support with CD (**Figure 8**). In our categorization, genes that passed all tests were considered primary targets (PP.H4 > 0.95), while genes that passed MR and SMR tests but had a PP.H4 less than 0.75 were categorized as tertiary targets. Genes that passed the MR and SMR tests and had a PP.H4 greater than 0.75 but less than 0.95 were classified as secondary targets.

**Table 2:** Gene and outcome co-localization results.

Disease	Gene	PP.H0	PP.H1	PP.H2	PP.H3	PP.H4	Grade
IBD	GPBAR1	0	0	0	0.01	0.99	Tier 1 target
	IL1RL1	0	0	0	0.05	0.95	Tier 1 target
	PRKCB	0	0	0	0.01	0.99	Tier 1 target
	PNMT	0	0	0	0.05	0.95	Tier 1 target
	FDPS	0	0.01	0	0.14	0.85	Tier 2 target
	RNASET2	0	0	0	0.22	0.78	Tier 2 target
	THBS3	0	0.14	0	0.47	0.52	Tier 3 target
UC	GPX1	0	0	0	0.02	0.98	Tier 1 target
	GPBAR1	0	0	0	0.01	0.99	Tier 1 target
	PNMT	0	0	0	0.01	0.99	Tier 1 target
	IGLC3	0	0.23	0	0	0.77	Tier 2 target
CD	IL1RL1	0	0	0	0.02	0.98	Tier 1 target
	GPR25	0	0	0.01	0.05	0.94	Tier 2 target
	FDPS	0	0	0	0.08	0.92	Tier 2 target
	STAT3	0	0	0	0.17	0.83	Tier 2 target
	END3	0	0.22	0	0	0.78	Tier 2 target
	ITGA4	0	0.45	0	0.03	0.52	Tier 3 target
	NDFIP1	0	0	0	0.86	0.14	Tier 3 target
	JAK2	0	0	0	0.99	0	Tier 3 target

IBD, Inflammatory bowel disease; UC, Ulcerative colitis; CD, Crohn’s disease.

PP.H0–PP.H4 represents the posterior probabilities of different hypotheses.

PP.H4 > 0.95 represents a strong colocalization between gene expression and risk of IBD, UC, and CD.

### 3.4 Estimation of druggability

We conducted a comprehensive search of the drug database for several genes identified in this study as potential drug targets (**Supplementary Table 7**). Our investigation revealed that targeting GPBAR1 is approved for the treatment of primary biliary cirrhosis, bile acid synthesis disorders, and various other diseases. Various drugs targeting PRKCB are associated with antioxidant effects, therapeutic benefits for relapsed glioblastoma multiforme, and preventive measures for vitamin E deficiency. Drugs targeting GPX1 exhibit multiple effects, including antioxidant activity and pain relief. However, no relevant information was found for IL1RL1 and PNMT as potential drug targets. Additionally, we explored secondary and tertiary targets identified in this study and found that drugs targeting FDPS have been approved for the treatment of osteoporosis. Similarly, drugs targeting ITGA4 are utilized for the treatment of multiple sclerosis, UC, and CD.

## 4 Discussion

In this study, we conducted a comprehensive investigation of 4302 genes to determine their association with the risk of IBD, UC, and CD. Using various MR methods (including Wald ratio/IVW, MR-Egger, Weighted median, Maximum likelihood, and MR-RAPS) and multiple sensitivity analyses (such as Cochran’s Q heterogeneity test, MR-Egger intercept pleiotropy test, and MR-Steiger directionality test), we explored target genes associated with the risk of IBD and its subtypes. To validate our results, we employed replicated MR, SMR, HEIDI tests, and multiple co-localization tests. Our comprehensive analyses revealed several significant findings. We found that the expression of the gene GPBAR1 was associated with an increased risk of IBD and UC. Conversely, the expression of PNMT was negatively associated with the risk of IBD and UC. Furthermore, higher levels of the genetically predicted gene IL1RL1 were linked to a reduced risk of IBD and CD. These findings provide valuable insights into the genetic factors influencing the risk of IBD and its subtypes, highlighting potential targets for further research and therapeutic interventions.

RNASET2 is the gene that encodes nuclease T2. It plays a key role in the intracellular context and its function involves processes such as RNA degradation and apoptosis. Prior work has identified RNASET2 as a susceptibility gene for IBD (30) and decreased RNASET2 expression has activating effects on pro-inflammatory cells, with an association with aggressive CD inflammation (31). The results of this study provide relatively strong evidence that RNASET2 levels may serve as an inflammatory biomarker for the prediction of progression in a novel disease.

The GPX1 gene is located at position 3p21.3 on the human genome and consists of five exons and four introns. The transcription product of this gene is a peptide containing 197 amino acid remnants that are translated into glutathione peroxidase 1 (GPX1) protein (32). GPX1 is mainly found in the cytoplasm of cells, where catalyzing the reaction between glutathione and substrates like hydrogen peroxide, decreases cellular damage by oxidative stress. Specifically, GPX1 uses reduced glutathione (GSH) to convert hydrogen peroxide to water and oxidizes GSH to oxidized glutathione (GSSG), which in turn generates GSH again via other reducing enzymes, maintaining the relative balance of GSSH and GSSG (33). Zhou et al. demonstrated the connection between endoplasmic reticulum stress-related genes and UC, and CD through a multi-omics approach and discovered that GPX1 expression lowered the risk of UC and CD (34). Oxidative stress leads to the inflammatory response exacerbated by oxidative damage to intracellular DNA, lipids, and proteins, which then triggers an inflammatory response in UC. GPX1 is known to be a toxicant through deleterious agents maintains redox balance, and can directly reverse the complex lipid peroxides in cells and tissues. It can also directly reduce complex lipid peroxides and minimize the damage of oxidative stress on cells and tissues. This may be the mechanism by which GPX1 reduces the risk of UC. Additionally, our study revealed that the gene GPX1 was relevant to UC, but did not find an association between GPX1 and CD. A study of 436 CD, 367 UC, and 434 controls showed that allele A in the gene GPX1 (rs1050450) was significantly observed to be associated with UC in a recessive model, and is a good candidate for a biological marker for the management of treatment of UC in the disease (35). An additional study using polymorphism-polymerase chain reaction in peripheral blood leukocytes from 1500 UC cases and 1500 healthy controls demonstrated that a genetic polymorphism in the GPX1 gene of 594TT is a danger factor for UC (36). They are consistent with the study we conducted, and future studies could explore the relationship of this gene with IBD as well as subtypes of CD to search for the more likely therapeutic targets.

STAT3 is an activator of signal transduction and transcription, playing a vital role within cells. Upon activation, STAT3 can enter the nucleus and regulate the transcription of several genes, thereby participating in the regulation of cell proliferation, apoptosis, inflammatory response, and other biological processes (37). According to a meta-analysis, the presence of the STAT3 rs744166 gene polymorphism may elevate the risk of developing CD, especially among Caucasians (38). A prior case-control study involving 232 CD patients and 272 controls indicated that the rs744166 and rs4796793 polymorphisms in the STAT3 gene may be linked to the onset of CD and are anticipated to serve as predictors of CD in the Chinese Han population (39). On one hand, STAT3 can regulate the activity of immune cells, such as macrophages and T cells, among others. It promotes the activation of immune cells and the release of inflammatory mediators while inhibiting the regulatory function of immune cells. This imbalance in the immune system leads to increased intestinal inflammatory response (40). On the other hand, IL-23 activates the STAT3 pathway, enhances the Th17 cell program, and contributes to the initiation and progression of pathological reactions (41). These findings align with the outcomes of the current study, suggesting that the STAT3 gene could serve as a potential therapeutic target for CD, warranting further clinical trials.

JAK2 is a tyrosine kinase that is involved in a variety of cytosolic signaling pathways, including apoptosis, differentiation, survival, and immune response (42). Drugs targeting JAK2 are considered for the treatment of immunological diseases such as UC, rheumatoid arthritis, and myelofibrosis. The present study found that JAK2 is a potential target for CD therapy. JAK2 signaling pathway can regulate the proliferation, differentiation, and activation of immune cells (like T-cells, B-cells, macrophages, etc.), and thus affects the normal function of the Immune system (43). Besides, alteration of intestinal barrier function would probably be one of the mechanisms by which JAK2 is involved in the pathogenesis of CD. One report of 464 CD patients, 292 UC patients, and 508 healthy controls in Germany revealed that patients carrying the C risk allele of the JAK2 rs10758669 gene polymorphism were at a higher frequency of increased risk of intestinal permeability (43). Targeted inhibitors of JAK2 have been studied and developed, and these drugs can interfere with the JAK2 modeling pathway and inhibit its aberrant activation, resulting in a reduction in the production of inflammatory mediators, alleviation of the inflammatory response, and amelioration of symptoms and disease progression in CD.

IBD is a complex group of diseases that includes multiple subtypes such as UC and CD. Despite sharing certain pathophysiological features, they differ dramatically in their clinical manifestations, histologic and immunologic features, and gene expression levels (44). Such differences point to the possibility that they may have different genetic mechanisms. Additional risk genes associated with IBD were identified in our survey, however, they are not currently validated by larger numbers of experimental studies. More studies may be needed to probe these genes in the future to prioritize IBD drug development. The strength of this study lies in its comprehensive screening of genes associated with IBD risk using the two-sample MR method, which effectively mitigates confounding bias. Moreover, the utilization of replicated MR, SMR, and co-localization to corroborate the experimental findings significantly bolsters the study’s conclusions, enhancing the robustness of the results and minimizing the potential for false positives. The evaluation of druggability offers promise for IBD treatment. However, there are several limitations to consider. Firstly, the genetic data were derived from a European population and necessitate further validation for extrapolation to other ethnic groups. Secondly, genetic regulatory mechanisms may exhibit tissue-specific variability, and focusing solely on blood eQTL may not afford a comprehensive understanding of the disease and its therapeutic avenues. Therefore, it is imperative to account for genetic regulatory diversity across multiple tissues and organs to gain a more nuanced comprehension of disease pathogenesis and identify effective treatments. Thirdly, while some genes associated with IBD risk have been experimentally validated, exploration of certain genes and their correlation with IBD risk remains deficient.

## 5 Conclusion

To summarize, our study employed sophisticated methods such as MR, SMR, and co293 localization to identify key genes intricately associated with the risk of IBD and its subtypes, UC and CD. Specifically, our analysis revealed the crucial roles of GPBAR1, IL1RL1, PRKCB, and PNMT genes in IBD pathogenesis while implicating GPX1, GPBAR1, and PNMT genes in UC susceptibility. Additionally, we found that IL1RL1 exhibits a protective effect against CD risk. These groundbreaking findings not only offer promising targets for the development of more effective biomarkers and therapeutic interventions but also deepen our understanding of the underlying molecular mechanisms driving IBD etiology. Nevertheless, further rigorous experimental and clinical investigations are required to validate and substantiate these findings before their translation into clinical practice.

## Data availability statement

The original contributions presented in the study are included in the article/**Supplementary Material**. Further inquiries can be directed to the corresponding author.

## Author contributions

SZ: Data curation, Formal analysis, Investigation, Methodology, Resources, Software, Validation, Visualization, Writing – original draft. YL: Conceptualization, Data curation, Formal analysis, Validation, Writing – review & editing. ZD: Conceptualization, Data curation, Investigation, Methodology, Visualization, Writing – review & editing.
